# Effect of Palm-Based Shortenings of Various Melting Ranges as Animal Fat Replacers on the Physicochemical Properties and Emulsion Stability of Chicken Meat Emulsion

**DOI:** 10.3390/foods12030597

**Published:** 2023-01-31

**Authors:** Mohd Razali Faridah, Masni Mat Yusoff, Ashari Rozzamri, Wan Zunairah Wan Ibadullah, Amelia Najwa Ahmad Hairi, Nur Hardy Abu Daud, Nurul Huda, Mohammad Rashedi Ismail-Fitry

**Affiliations:** 1 Department of Food Technology, Faculty of Food Science and Technology, Universiti Putra Malaysia, Serdang 43400, Malaysia; 2 Department of Food Science, Faculty of Food Science and Technology, Universiti Putra Malaysia, Serdang 43400, Malaysia; 3 Sime Darby Plantation Research Sdn. Bhd., Lot 2664, Jalan Pulau Carey, Petaling Jaya 42960, Malaysia; 4 Faculty of Sustainable Agriculture, Universiti Malaysia Sabah, Sandakan 90509, Malaysia; 5 Halal Products Research Institute, Universiti Putra Malaysia, Serdang 43400, Malaysia

**Keywords:** fat mimetics, fat substitutes, meat products, palm-based products, sausages

## Abstract

This study evaluated the effects of palm shortenings (PS) with varying melting ranges (MR) on the physicochemical, emulsion stability, rheological, thermal, textural, and microtextural properties of chicken meat emulsions. Six emulsions were developed: control (chicken skin), sample A (PS at MR of 33–36 °C), sample B (PS at MR of 38–42 °C), sample C (PS at MR of 44–46 °C), sample D (PS at MR of 45–49 °C), and sample E (PS at MR of 55–60 °C). There were no significant differences in cooking loss, pH, and water-holding capacity between the meat emulsions, with sample E providing a more stable emulsion with the lowest fat content and highest moisture content. The colour profiles and protein thermal stabilities of the fat-replaced meat emulsions were not significantly different from the control. The hardness, shear force, storage, and loss moduli increased when palm shortenings with higher melting range were used, with sample E having the highest values. Sample E also exhibited a smaller pore size and more compact structure, and thus was well-emulsified compared to the other samples. Overall, palm shortenings–particularly those with a melting range of 55–60 °C–have the potential to replace chicken skin in meat emulsions.

## 1. Introduction

A meat emulsion is a complex oil-in-water emulsion system where fat acts as the dispersed phase, while an aqueous matrix system containing water, salt-soluble proteins, muscle fibres, connective tissues, collagen, and other ingredients acts as the continuous phase, and protein acts as the emulsifier [1,2,3]. A stable meat emulsion with excellent functional properties, including texturization and holding of fat, water, and particles, is a base for producing well-emulsified meat products such as sausages, frankfurters, meatballs, meatloaf, and bologna [1,4]. Fat is a significant ingredient as it stabilises the meat emulsion, improves the rheological, viscosity, textural, and structural properties, as well as provides a distinctive taste profile [5,6]. It is also an important source of energy, fat-soluble vitamins (A, D, E, and K) and essential fatty acids [7], but too much animal fat is unhealthy as it contains a substantial amount of saturated fats, trans fats, and cholesterol, which are associated with coronary heart disease, hypertension, cardiovascular disease, and obesity [8]. 

Palm oil is an excellent source of vitamin E as well as tocopherols and carotenoids–natural antioxidants that protect against oxidative deterioration [9]. Palm oil products such as solid palm stearin and liquid palm olein have been used in various food applications due to their unique fatty acid and triacylglycerol (TAG) profile [10] and are easily incorporated into various food products without the need for hydrogenation [11]. The hydrogenation of oils causes the production of trans fats [12], which decrease the ‘good’ cholesterol (high-density lipoproteins) while increasing the ‘bad’ cholesterol (low-density lipoproteins)–which is directly linked to the risk of cardiovascular diseases [13]. Therefore, natural lipid sources and lipids that undergo minimal processing procedures are preferred in food applications.

Shortenings are 100% fat products derived from animal fats or vegetable oils [14] that tenderise foods by interrupting or shortening the fat films that form during heating, thereby preventing the hardening of carbohydrate and protein components [15]. Palm-based shortenings do not require hydrogenation due to the natural state of the palm oil that contains a high degree of shorter-chain saturated fatty acids, which incorporates the liquid components into the crystal network, thereby providing the shortening structure and texture [16]. Furthermore, palm oils have been widely used to replace hydrogenated lipids in solid-fat formulations and high-fat food due to their high solid-fat content at ambient temperatures, which contributes to the stability and structure, and long shelf-life (due to high oxidative stability) [14,17].

Most shortenings are formulated for their application in specific food products, with the melting profile being one of their most crucial physicochemical properties. The melting profile is the temperature at which the lipid changes from a solid to a liquid state and is dependent on the nature of its fatty acid components [18,19], therefore, the term ‘melting ranges’ is more suitable to explain the melting profile of fats, oils, and their by-products as they are a mixture of different triacylglycerols [20]. The shortenings can be tailor-made by altering the degree of saturation and the length of the hydrocarbon chain by rearranging the fatty acids [15,21]. Theoretically, palm oil has the potential to be employed as a raw material in the production of shortening due to its triacylglycerol composition [22] and higher melting temperature than most other plant oils [14].

Several studies have reported the promising results of substituting animal fats with palm oils, for example, Wang et al. [23] substituted pork back fat with palm oil Pickering emulsion to produce a more homogenous microstructure, improved viscoelastic response and textural properties, less cooking loss, and brighter colour of emulsified pork sausages. Wan Rosli et al. [24] reported that the substitution of beef fat and chicken fat with red palm oil improved the nutritional quality by increasing the vitamin E content without affecting other physicochemical properties of burger patties. As described earlier, palm shortenings of specific melting ranges can be developed for specific food applications. Therefore, this study evaluated palm shortenings of varying melting ranges (33–36 °C, 38–42 °C, 44–46 °C, 45–49 °C, and 55–60 °C) as animal fat replacers and determined their effects on the physicochemical properties, emulsion stability, thermal, rheological, and textural and microtextural properties of chicken meat emulsion.

## 2. Materials and Methods

### 2.1. Preparation of Meat Emulsion

Five palm shortenings with different melting ranges were supplied by Sime Darby Research Sdn. Bhd. The palm shortenings were labelled as A (MR of 33–36 °C), B (MR of 38–42 °C), C (MR of 45–49 °C), D (MR of 44–46 °C), and E (MR of 55–60 °C). The palm shortenings were kept in airtight containers and stored in a chiller at 4 °C until further usage. Chicken breast meat, chicken skin, salt, and sodium tripolyphosphate (STPP) were purchased from Sri Ternak Mart, Seri Kembangan, Selangor. The meat emulsions were prepared following the procedures described by Ismail et al. [25] with slight modifications. The chicken breast and chicken skin were rinsed with clean water before being ground separately in a mincer machine (Model 4822, Hobart, Offenburg, Germany. The control meat emulsion was formulated with 70% chicken meat, 15% fat, 1% salt, 0.5% STPP, and 13.5% ice water (Table 1). 

For the treatment of fat-replaced samples, the chicken skin was replaced by palm shortenings with varying melting ranges at 15% of the total weight of the formulation. The ground meat, salt, phosphate, and half of the ice water were placed into a food processor (Model MK-5087M-NS, Panasonic, Selangor, Malaysia) and mixed for 30 s. Then, the ground chicken skin or palm shortenings was added as the fat replacer, and further mixed for 15 s. The remaining ice water was added and mixed for another 20 s until the emulsion was fully homogenized. The meat emulsion was weighed to approximately 40 g and transferred into a centrifuge tube. The stuffed centrifuge tubes were centrifuged (Model 3740, Kubota, Fujioka, Japan) at 2500 rpm at 3 °C for one minute to eliminate air bubbles. The uncooked meat emulsion was kept frozen in a freezer at −18 °C until further analysis.

### 2.2. Emulsion Stability

The stability of the meat emulsion was evaluated based on the total expressible fluid and fat using the method of Ismail et al. [25], with some modifications. First, 15 g of the uncooked sample was stuffed into a labelled centrifuge tube and centrifuged at 3000 rpm at 4 °C for 15 min and heated in a water bath (Waterbath WNB 14, Memmert, Schwabach, Germany) for 30 min at 75 °C. The lid of the centrifuge tubes was open and left upside down in labelled crucibles for 1 h to drip dry the expressible fluid. The palleted samples were weighed, while the supernatants obtained were oven-dried overnight at 105 °C. The volumes of total expressible fluid (TEF) and the expressible fat (EFAT) were calculated using the formula as follows:

TEF = (weight of centrifuge tube + weight of sample) − (weight of centrifuge tube + weight of pellet)(1)

%TEF = TEF/(sample weight) × 100(2)

%EFAT = [(weight of crucible + weight of dried supernatant) − (weight of empty crucible)]/TEF × 100(3)

### 2.3. Cooking Loss

The cooking loss was determined using the procedures provided by Ismail et al. [25] with minor modifications. First, 5 g of each uncooked meat emulsion sample was weighed and deposited into a centrifuge tube, then centrifuged at 1000× *g* for 4 s to eliminate air bubbles. The samples were then immersed in a 50 °C preheated water bath and the temperature was gradually increased until the samples achieved an internal temperature of 72 °C for 15 min, as measured by a thermocouple. The centrifuge tubes were cooled in a cold-water bath for 5 min, then the exudates were discharged, and the cooked samples were removed from the centrifuge tubes and wiped dry using filter paper. The cooked samples were weighed, and the results were calculated as follows:

Cooking loss = (weight of uncooked sample − weight of cooked samples)/(weight of uncooked sample) × 100(4)

### 2.4. pH

The pH values of cooked meat emulsion samples were measured using a pH meter (Eutech pH 2700, Singapore) equipped with a pH electrode according to Jauhar et al. [26]. Then, 5 g of each cooked sample was homogenized with 20 mL of distilled water before measurement.

### 2.5. Water Holding Capacity (WHC)

Water holding capacity can be defined as the ability of the meat emulsion to retain water during the application of outside forces such as centrifugation. The procedure of WHC determination was completed according to the method described by Köhn et al. [27]. First, 5 g of uncooked samples were manually mixed with 32 mL of distilled water in a centrifuge tube and left for 10 minutes. The samples were then centrifuged at 2900 rpm for 25 min using a refrigerated micro centrifuge (Model 3740, Kubota, Fujioka, Japan). The supernatants were discarded, and the samples were dried in an oven at 50 °C for 20 min with a 10–20° inclination downwards. The WHC was calculated using the following equation:

WHC = [(b − a) − (c − a)]/(b − a)(5)

where,

a = weight of empty centrifuge
b = weight of centrifuge with supernatant
c = weight of dried centrifuge

### 2.6. Texture Profile Analysis

The texture profile of the cooked meat emulsion samples was analysed using a Texture Analyser TA-XT2 (Stable Micro Systems, Surrey, UK) following the method described by Ismail et al. [28]. The cooked samples were cut into a cylindrical shape of 10 mm length × 20 mm diameter and compressed twice using a P/75 probe equipped with a 30 kg load to 75% of the original height. The texture analyser was set with a test speed of 1.5 mm/s and a post-test speed of 1.5 mm/s during the measurement. The parameters assessed were hardness, cohesiveness, gumminess, and chewiness.

### 2.7. Shear Force Test

The measurement of the Warner-Bratzler shear test followed a method described by Ismail et al. [28] with some proper modifications implemented. The cooked meat emulsion samples of 25 mm length × 15 mm diameter were placed horizontally and cut by using a texture analyser (Model TA-XT2i, Stable Micro System, Surrey, UK) attached with a Warner-Bratzler (WB) shear blade with a triangular slow cutting edge (1 mm of thickness) at a cut speed of 1.5 mm/s. The maximum shear force (N) and work of shearing (N·s) were obtained.

### 2.8. Proximate Analysis

The proximate compositions of chicken skin and cooked meat emulsion samples were determined following the Official Methods of the Association of Official Analytical Chemists [29]. Moisture content was evaluated by drying the sample in the oven at 105 °C overnight and the weight difference between the sample before and after drying was calculated. The ash content was determined by burning the dried sample in a furnace at 550 °C (Method No. 930.05). The crude protein was determined following the micro-Kjedhal method (Method No. 978.04), where the nitrogen conversion factor used was 6.25. The crude fat was obtained by using the Soxhlet extraction method, where petroleum ether was used as the extraction solvent at 70 °C for 8 h and the weight of fat extracted from the chicken skin and cooked samples in the petroleum ether was measured (Method No. 930.09).

### 2.9. Colour Analysis

Colour measurements for cooked meat emulsion samples were examined according to Ismail et al. [28]. The cooked sample was thinly cut and arranged in a 5 cm diameter of transparent plastic container. The surface colour of the samples was evaluated for the rates of lightness (L*), redness (a*), and yellowness (b*) by using a hand-held chromameter (CR-40, Minolta Camera Co., Japan) with an aperture size of 8 mm.

### 2.10. Rheological Properties

The dynamic rheological analysis of uncooked meat emulsion samples was conducted by adapting the methods described by Kim et al. [30] with some modifications. The procedure was carried out by using a rheometer (Anton paar Rheometer, MCR 302 Series, Austria) equipped with a 35 mm diameter stainless steel plate with a 1 mm size gap. The samples were carefully placed onto the plate using a plastic spoon and left to be equilibrated to a temperature set at 25 °C for 5 min. A stress sweep test (0.1–100 Pa at 1 Hz frequency) was conducted prior to the frequency sweep test to determine the linear viscoelastic region (LVR). The frequency sweep test was performed at 0.5% strain, which was within the LVR with a frequency range of 0.1–10 Hz. The results were expressed as storage modulus (G′) and loss modulus (G″), and the loss factor tangent (tan = G′′/G′) was determined. Additionally, a temperature sweep test was conducted in the temperature range of 25–75 °C at a constant heating rate of 2 °C/min. The changes in G′ values were recorded during the measurements.

### 2.11. Differential Scanning Calorimetry (DSC)

The thermal properties of uncooked meat emulsion samples were determined using the procedures described by referring Kim et al. [30], with some modifications. In brief, 7 mg of samples were placed in an aluminium pan, hermetically sealed, and analysed between 30 and 100 °C at a rate of 10 °C/min. An empty pan was used as a reference for all analyses.

### 2.12. Scanning Electron Microscopy

The microstructures of the cooked meat emulsion samples were observed using a variable pressure scanning electron microscope (Model 1455 VPSEM, Leo/Zeiss, Cambridge, UK) as described by Ismail et al. [28]. The sample (5 mm thick) was fixed with 0.1 mol/L phosphate buffer (pH 7.0) containing 2.5% glutaraldehyde at 4 °C for 24 h and dehydrated in an incremental concentration of ethanol solution (50, 70, 90, 95 and three times with 100%) for 10 min per solution. Then, the samples were freeze-dried in acetone cooled in liquid nitrogen, mounted over the stubs using a double-sided carbon conductivity tape and sputter-coated with gold for 3 min using an automated sputter coater. The specimen was observed at the magnification of 250 until 2000×.

### 2.13. Statistical Analysis

All analyses were done in triplicate for each of the meat emulsion samples. Collected data were analysed by using Minitab Statistical Software Version 19 (Minitab Inc., State College, PA, USA). One-way analysis of variance (ANOVA) and Tukey’s test for pairwise comparison between the six samples were carried out with a confidence level of 95% (α = 0.05) for all the analyses.

## 3. Results and Discussion

### 3.1. Emulsion Stability, Cooking Loss, pH and Water Holding Capacity

The stability of the test emulsions is expressed as total expressible fluid (%TEF) and the expressible fat (%EFAT), with a more stable meat emulsion having a lower %TEF and %EFAT [31]. As shown in Table 2, sample E had a significantly lower %TEF compared to the other samples, while no statistical difference was observed for %EFAT. Theoretically, the higher the melting range, the higher the degree of saturation; thus, the palm shortening used in sample E, which has the highest melting range/degree of saturation compared to the other samples, produced a more stable emulsion since the amount of fluid released from the emulsion structure was substantially small, while the amount of fat released was comparable to the emulsion made with chicken skin. A lower melting temperature or an increase in the unsaturation of fats results in weak emulsion stability, causing losses and poor texture of sausages (a product of the meat emulsion system) [32].

The cooking loss (and cooking yield) is determined by the capability of the protein matrix to immobilize water and fat [33]. According to Table 2, the cooking loss of the test emulsions was not significantly different from the control. Similarly, Youssef and Barbut [6] showed no significant differences in the cooking loss of meat emulsions prepared with hydrogenated palm oil, and Hsu and Yu [34] reported no significant differences in cooking yield between emulsified Kung-wan (Taiwanese-style meatballs) made with pork back fat and hydrogenated vegetable oils. Kumar [33] explained that animal fat particles in meat products can function as a barrier to water, thereby trapping the water inside the protein matrix. Taken together, these data indicate that palm shortening and other hydrogenated vegetable oils are comparable to animal fats in terms of their ability to act as water barriers that positively affected cooking losses of meat emulsions.

Regarding the pH, there were no significant differences in the pH between the control and test emulsions (Table 2), indicating that the different melting temperatures of palm shortening minimally influenced the acidity of the meat emulsions. Furthermore, previous studies also showed no notable differences in the pH of emulsified meat products prepared with various types of lipid-based fats [23,35,36]. 

The water holding capacity (WHC) is defined as the ability of the meat to retain moisture with the influence of outside forces [31], which is a crucial parameter to determine the emulsion stability. As shown in Table 2, there was no significant difference in the WHC observed for all meat emulsion samples. Álvarez et al. [37] reported that the WHC is inversely related to water loss or cook loss, however, there was no significant difference in cooking loss and WHC between the meat emulsions tested in this study. Youssef and Barbut [6] also used rendered beef fat and palm oil in meat emulsions and reported no notable differences in fluid/water loss, which was most likely due to similarities in their physicochemical characteristics.

### 3.2. Textural Properties and Shear Force Test

Table 3 shows the textural properties and shear force results of the meat emulsion samples. Sample E presented the highest hardness (*p* < 0.05) compared to the other samples, while the maximum shear force and work of shearing were comparable to the control sample but higher than the other fat-replaced samples. The shortening used in sample E has the highest melting range, and thus the highest degree of saturation, which further contributed to its hardness. Therefore, the incorporation of this shortening simultaneously resulted in the highest hardness of sample E. When the fat hardness increased, the comprehensive force remarkably increased [38]. Youssef and Barbut [6] showed that the incorporation of hydrogenated palm oil in meat emulsion resulted in the highest hardness since hydrogenated palm oil is a hard fat, and its inclusion in the protein matrix enhanced the compression resistance of the meat emulsion. This might also explain why the high hardness of the shortening used in sample E increased the resistance of the cooked meat emulsion to be sheared, thus increasing the maximum shear force. A possible explanation for the control sample demonstrating a statistically higher maximum shear force and work of shearing might be due to the presence of chicken skin connective tissues, which are mainly collagen and turn into gelatin when heated, thus contributing to a firmer texture [39]. 

The springiness of the meat emulsion samples was significantly lower than the control sample, except for sample D and sample E. The cohesiveness value was highest in the control sample than that of the other fat-replaced samples, except for sample D. The chewiness of sample E was the highest and is one of the textural parameters generated by hardness, thus accounting for the similar pattern of chewiness and hardness. Overall, sample D showed the most compatible texture profile properties compared to the control sample.

### 3.3. Proximate Composition and Colour Properties

Table 4 shows the proximate composition results that were in line with the data recorded by Choi et al. [40]. All fat-replaced samples exhibited significantly lower moisture content than the control sample. Chicken skin contains about 53.10% moisture, whereas the other palm shortenings used as fat replacers in this study have approximately 0% moisture content, thus, the chicken skin used as fat in the control sample might contribute to the high moisture content in this sample. Sample E had the highest moisture content among all the fat-replaced samples. During cooking when the meat emulsions were subjected to heat treatment, the fat starts to melt, causing an increase in fat liquefication that leads to a reduction in water binding capacity [41]. In the current study, the meat emulsion was cooked until the sample reached an internal temperature of 72 °C, the temperature at which all fats were considered to have converted to the liquid phase. However, considering the fat incorporated in sample E has the highest melting range resulting in the highest moisture content among the fat-replaced samples, it can be concluded that the higher the melting temperature of fats, the better the water binding capacity in the meat emulsion system.

The use of palm shortenings as fat substitutes significantly increased the fat content of all emulsions tested, except for sample E that had a lower fat content compared to the control. A lower fat content for the control sample was expected because chicken skin has a lower fat content (31.69%) than palm shortenings (100%). The lower fat content of sample E might be due to the wide dispersion of smaller fat globules (see Section 3.6), which reduces the free distance between the fat globules, causing them to clump together and leach out during cooking [42]. 

There were no significant differences in the protein and ash contents of all emulsions. Chicken skin comprises about 13.66% protein, while fat shortenings do not contain any protein. However, the protein content in chicken skin is considered low as it does not significantly increase the total protein content of the control compared to fat-replaced emulsions. No notable difference was observed in ash content due to the low mineral content of all ingredients used to produce the meat emulsions, which was in line with other literature reporting the substitution of animal fats with lipid-based fat replacers in meat emulsions [43,44].

**Figure 1:**
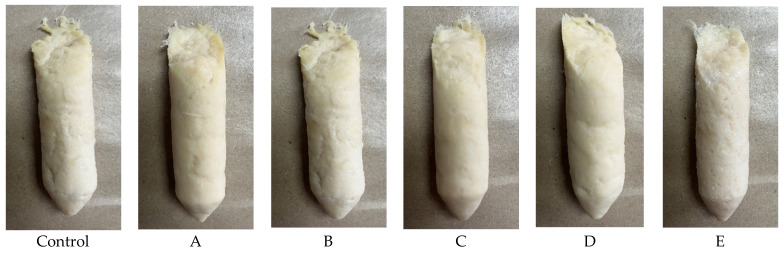
The images of cooked fat-replaced samples with palm shortenings (PS) of various melting ranges (MR) as compared to the control sample with chicken skin. **Control** (chicken skin), sample **A** (PS at MR of 33–36 °C), sample **B** (PS at MR of 38–42 °C), sample **C** (PS at MR of 44–46 °C), sample **D** (PS at MR of 45–49 °C), and sample **E** (PS at MR of 55–60 °C).

The colour properties of the emulsions are presented in Table 4, showing that the different melting ranges of palm shortenings significantly affected the lightness (L*) and redness (a*) but not the yellowness (b*) of the emulsions. The L* values of all fat-replaced samples were higher than those of the control, however, only samples C and E were significantly higher. This result is in good agreement with the results of Wang et al. [23], who found the incorporation of palm oil Pickering emulsion in emulsified sausage resulted in higher L* values. Youssef and Barbut [45] found a similar result when replacing beef fat with canola oil in meat batters; they concluded that smaller fat globules of canola oil provide a larger surface area, hence more light was reflected from the sliced surface. Although there were significant differences between the control sample and samples D and E in terms of a* values, the a* values for all meat emulsion samples are considered low (2.42–3.57). This might be due to the low myoglobin content in chicken meat, which gives the meat products their red colour [46]. There was no significant difference in yellowness (b*) between meat emulsion samples prepared with chicken skin or palm shortenings, probably due to the original colour of the palm shortenings not being much different from the colour of the chicken skins. In addition, all cooked fat-replaced samples exhibited visually identical colours to the control (Figure 1), thus, replacing chicken skin with palm shortenings does not raise any concern in terms of colour emulation.

### 3.4. Rheological Properties 

#### 3.4.1. Frequency Sweep

**Figure 2:**
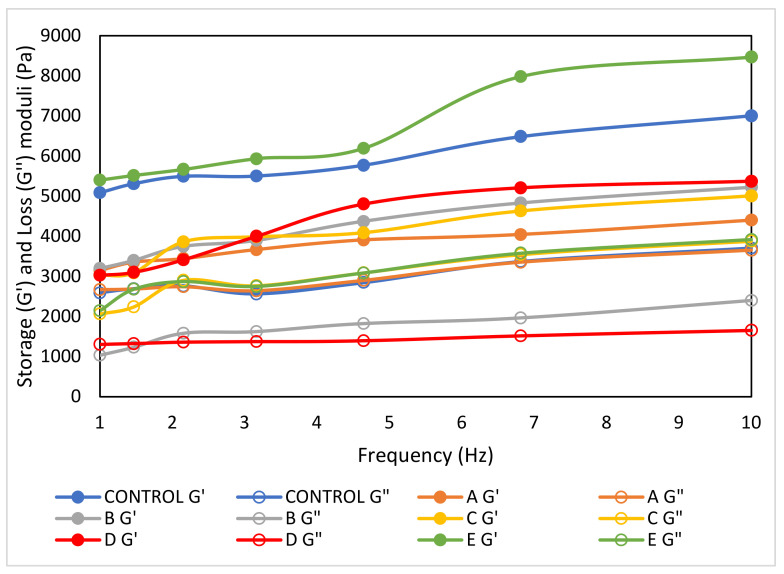
Storage (G′) and loss (G″) moduli of fat-replaced samples with palm shortenings (PS) of various melting ranges (MR) as compared to the control sample with chicken skin. Control (chicken skin), sample A (PS at MR of 33–36 °C), sample B (PS at MR of 38–42 °C), sample C (PS at MR of 44–46 °C), sample D (PS at MR of 45–49 °C), and sample E (PS at MR of 55–60 °C).

Figure 2 illustrates the changes in storage (G′) and loss (G″) moduli values as a function of frequency, showing that the G′ values were higher than the G″ values, indicating that the samples were more elastic than viscous and show a common viscoelastic characteristic of a typical ‘weak gel’ [47]. Similar results were acquired for emulsified sausages containing β-cyclodextrin/konjac-based emulsion gel as a fat substitute [48], low-fat meat emulsion containing aloe gel [47], and fat-substituted sausage containing inulin as a fat substitute [49].

Sample E–which had the highest melting range of shortening–possessed the highest G′ and G″ values and showed small proportional differences in the G and G″ values with frequency over a 1–10 Hz range, suggesting that it had a well-organized matrix structure [48], thus a more stable emulsion. In comparison to the other samples, sample E displayed a higher G′ value at a lower frequency with a greater discrepancy between G′ and G″, indicating the strongest network structures among all samples. This result agreed with the findings reported by Kim et al. [30] and Kim et al. [50], who observed that the samples incorporated with solid fats had higher melting temperatures resulting in higher G′ and G″ values compared to the samples that used liquid fats (lower melting temperatures). In the current study, the physical state of the palm shortening used in sample E exists in a solid state, whereas the other palm shortenings exist in semi-solid forms at the analysed temperature (25 °C), suggesting that a higher degree of saturation of fat resulted in a better rheological property.

**Figure 3:**
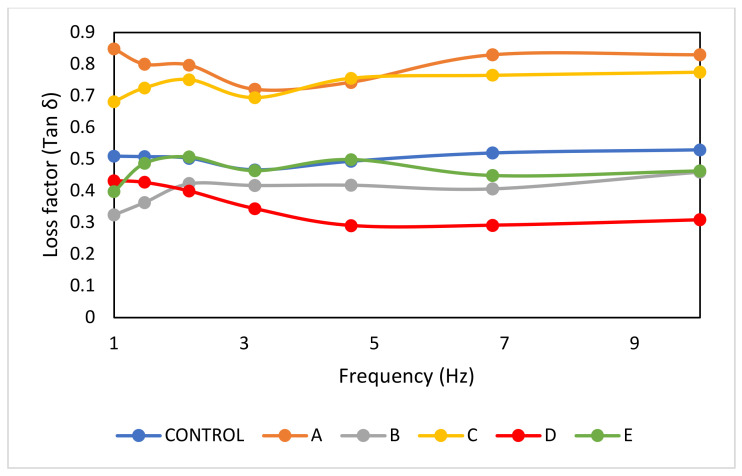
Loss factor (tan δ) of fat-replaced samples with palm shortenings (PS) of various melting ranges (MR) as compared to the control sample with chicken skin. Control (chicken skin), sample A (PS at MR of 33–36 °C), sample B (PS at MR of 38–42 °C), sample C (PS at MR of 44–46 °C), sample D (PS at MR of 45–49 °C), and sample E (PS at MR of 55–60 °C).

In addition, a higher storage modulus also indicates a stronger gel and more rigid structure [51] due to the formation of a stabilized network structure or strong particle–particle interactions [52]. It has been suggested that a loss factor value lesser or higher than 1 is indicative of gel-like behaviour or sol-like behaviour, respectively [53]. As shown in Figure 3, the loss factor values for all samples showed values less than 1, thus the samples exhibited more gel-like behaviour. Kumar [47] mentioned that the higher tan δ values resulted in a more stable matrix. Although the highest tan δ value was observed for sample A, due to the insignificant difference in the tan δ values of all samples, sample E was still considered a stable matrix emulsion.

#### 3.4.2. Temperature Sweep

A temperature sweep was conducted to observe the gelling ability and gelation point of meat emulsion subjected to fats at various melting ranges. The shift in the G′ values was mainly caused by the denaturation of the myofibrillar protein during the heating process, indicating that the physical state of the emulsion transforms from a highly viscous to a highly elastic state as the temperature rises [54]. This state transition also suggests that the meat emulsion changed from a weak network to a more organised gel matrix. 

**Figure 4:**
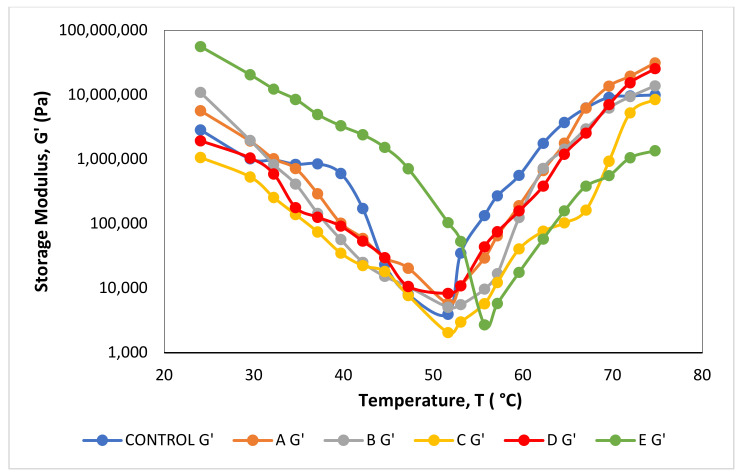
Storage modulus (G′) of fat-replaced samples with palm shortenings (PS) of various melting ranges (MR) as compared to the control sample with chicken skin as a function of temperature. Control (chicken skin), sample A (PS at MR of 33–36 °C), sample B (PS at MR of 38–42 °C), sample C (PS at MR of 44–46 °C), sample D (PS at MR of 45–49 °C), and sample E (PS at MR of 55–60 °C).

According to Figure 4, the G′ values for all samples encountered a substantial drop at around 25–50 °C, and then drastically increased from 51 °C upward. This observation matched the gelation theory of myosin which occurs in two stages: the first occurs between 30 °C and 50 °C when the globular heads of myosin begin to aggregate, and the second occurs above 50 °C when structural changes to the myosin tail’s helix form take place [55]. A surge of G′ value for the second time at temperatures above 50 °C subsequently due to the production of a stable three-dimensional network structure and permanently irreversible cross-linked myosin filaments [56]. According to the literature, the gelation point of meat emulsion is stipulated at the lowest magnitude of G′ values [54]. Correspondingly, the rheograph showed that the gel formation temperature of the meat emulsion was around 52 °C, except for sample E which showed a higher gelation point at 56 °C (Figure 4), indicating that sample E needed more heat to encourage gelling and create a gel structure. A higher gelation point also suggests that greater thermal energy is required to break the bonds that lead to protein deformation and aggregation, denoting that the palm shortening incorporated in sample E could encourage a complete protein–protein interaction. According to Ashari [56], a higher amount of thermal energy for gelation is needed to dissociate a more stable protein network. This implies that palm shortening at a melting range of 55–60 °C could prevent protein denaturation during the freezing period, thus persevering the protein in its ideal state. 

### 3.5. Differential Scanning Calorimetry (DSC)

According to Kazemi et al. [57], DSC can be used to study the thermal properties of the complex food system and its components such as water, protein, and fat in response to different temperatures. The DSC results of the meat emulsion samples showed two peaks: peak 1 (fats melting point), and peak 2 (protein denaturation point) (Table 5). However, this result was not in line with that reported by Kim et al. [30], who observed three peaks–the other peak was described as the moisture thawing point. In the current study, no thawing point was observed since the DSC analysis was performed at 30–100 °C and the meat emulsions were thawed to room temperature before analysis, whereas the DSC analysis by Kim et al. [30] started at −60–100 °C.

When comparing the data for peak 1 (Table 5), all fats incorporated in meat emulsions exhibited significantly different onset, peak, and end temperatures, where the data represent the melting profile of each fat. Sample E showed the highest onset, peak, and end temperatures, followed by sample D, sample C, sample B, control, and sample A. The melting temperature of chicken skin fat ranged from 35.01–37.28 °C. The enthalpy data also showed a similar trend, which increased as the melting temperatures of the fats increased. This trend was explained by the differences in saturated/unsaturated ratios of the fatty acids contained in the chicken skin and palm shortenings, as these have a notable impact on the enthalpy and melting point of the fats [58]. As the melting temperature of the fats increases, they are composed of more saturated and less unsaturated fatty acids, thereby requiring more energy to reach the highest peak. Therefore, sample E recorded the highest enthalpy value followed by the samples incorporated with lower melting temperature fats. Kim et al. [30] also mentioned that a higher enthalpy was due to the difference in the heat conductivities of the fats, whereby a harder (solid) fat required more energy to change its phase to the liquid state compared to softer (semi-solid or liquid) fats.

The data for peak 2 (Table 5) reveal a minimum effect of shortenings and chicken skin on protein thermal denaturation temperature of chicken meat emulsions regardless of the different ranges of melting temperature. There were no significant differences in onset temperatures, peak temperatures, end temperatures, and enthalpy observed in all meat emulsion samples. The data also showed common protein denaturation temperatures of chicken meat protein as reported in the literature [59,60,61]. This result might be explained by the fact that the fats in all the emulsions were entirely melted at the temperature at which the protein began to denature (around 62 °C). Thus, no solid fat particles present in the meat emulsion at this condition could hinder the process of protein thermal denaturation. Since there was no significant difference observed for peak 2, it can be suggested that palm shortenings with varying melting ranges are comparable to replacing chicken skin in meat emulsion when focused on the protein thermal stability.

### 3.6. Scanning Electron Microscopy (SEM)

**Figure 5:**
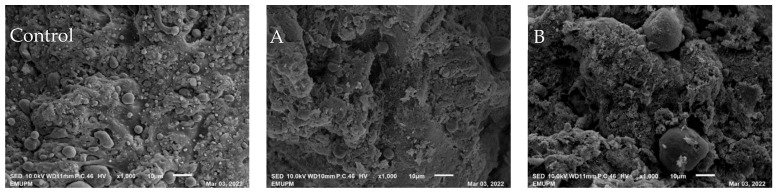

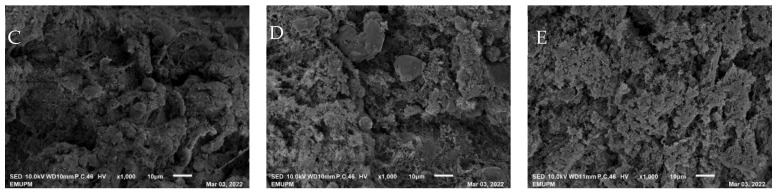
SEM photomicrograph of fat-replaced samples with palm shortenings (PS) of various melting ranges (MR) as compared to the control sample with chicken skin. (control—e: 1000 × magnification). **Control** (chicken skin), sample **A** (PS at MR of 33–36 °C), sample **B** (PS at MR of 38–42 °C), sample **C** (PS at MR of 44–46 °C), sample **D** (PS at MR of 45–49 °C), and sample **E** (PS at MR of 55–60 °C).

The SEM micrographs (Figure 5) reveal the three-dimensional features of common heat-induced meat emulsions where the fat globules were embedded and encapsulated in the protein matrix. These results were in good agreement with previous studies [35,62]. All meat emulsion samples show fat globules of varying sizes and irregular round to oval shapes (Figure 5). The control sample shows the most obvious fat globules compared to those samples incorporated with palm shortenings. The protein matrix had various sizes of pores, which according to Shin et al. [35] are responsible for capillary action that helps to hold water inside the heated meat emulsion systems. Ismail et al. [28] reported that a smaller pore size indicates a more stable emulsion. Sample E (Figure 5E) with the highest melting range of palm shortening had smaller pores and a more compact structure, therefore, it was considered the most well-emulsified. The fat globules in sample E could not be clearly detected due to their smaller size and dispersion so it was difficult to differentiate the pores within the protein matrix. An intact and compact morphological structure shown by sample E (Figure 5E) might explain the higher hardness than other samples (Table 2).

## 4. Conclusions

In conclusion, replacing chicken skin with palm shortening produces a stable meat emulsion system. The incorporation of a higher melting range (55–60 °C) palm shortening results in a well-emulsified meat emulsion with low-fat content and better moisture content and gelation ability, and was comparable to the chicken skin control in terms of cooking loss, pH, water holding capacity, and protein thermal stability. However, the presence of palm shortening in form of a solid-state–despite having the highest melting range–resulted in a harder texture, which was also supported by fat globules distribution and protein matrix structure shown by SEM micrographs. Overall, palm shortening at a melting range of 55–60 °C, which was incorporated in sample E, could be considered the best palm shortening used to replace chicken skin in producing a stable meat emulsion, while palm shortenings incorporated in sample C (MR of 44–46 °C) and sample D (MR of 45–49 °C) could be considered the second-best palm shortenings to replace chicken skin in meat emulsion as they showed comparable results with the control and did not detrimentally affect the textural properties of the cooked meat emulsion. These findings could be the basis for future research on the development of emulsified meat-based products with a lower animal fat content.

## Figures and Tables

**Table 1:** Formulations of the fat-replaced meat emulsion samples with palm shortenings (PS) of various melting ranges (MR) as compared to the control with chicken skin.

Ingredients	Amount (%)	Samples (g/200 g)					
		Control	A	B	C	D	E
Chicken breast meat	70	140	140	140	140	140	140
Fat	15	-	-	-	-	-	-
Chicken skin	-	30	-	-	-	-	-
PS 33–36 °C	-	-	30	-	-	-	-
PS 38–42 °C	-	-	-	30	-	-	-
PS 44–46 °C	-	-	-	-	30	-	-
PS 45–49 °C	-	-	-	-	-	30	-
PS 55–60 °C	-	-	-	-	-	-	30
Salt	1	2	2	2	2	2	2
Sodium Tripolyphosphate (STPP)	0.5	1	1	1	1	1	1
Ice Water	13.5	27	27	27	27	27	27
Total	100	200	200	200	200	200	200

**Table 2:** The cooking loss, pH values, and water-holding capacity of the fat-replaced samples with palm shortenings (PS) of various melting ranges (MR) as compared to the control sample with chicken skin.

Samples	TEF (%)	EFAT (%)	Cooking Loss (%)	Ph Raw	WHC (%)
Control	16.37 ± 1.06 ^abc^	8.13± 0.51 ^a^	21.83 ± 3.17 ^a^	6.33 ± 0.06 ^a^	89.74 ± 0.28 ^a^
A	16.44 ± 2.13 ^ab^	11.96 ± 2.67 ^a^	28.67 ± 3.06 ^a^	6.30 ± 0.00 ^a^	91.20 ± 0.24 ^a^
B	18.85 ± 0.95 ^a^	14.02 ± 2.79 ^a^	30.87 ± 1.03 ^a^	6.27 ± 0.06 ^a^	90.80 ± 0.22 ^a^
C	12.19 ± 1.60 ^c^	11.05 ± 1.49 ^a^	23.22 ± 4.86 ^a^	6.27 ± 0.00 ^a^	91.30 ± 2.58 ^a^
D	13.34 ± 2.04 ^bc^	8.19 ± 2.10 ^a^	27.12 ± 6.21 ^a^	6.37 ± 0.06 ^a^	90.35 ± 0.37 ^a^
E	5.09 ± 1.03 ^d^	13.43 ± 2.89 ^a^	25.46 ± 7.92 ^a^	6.30 ± 0.00 ^a^	90.02 ± 0.22 ^a^

Means followed by different uppercase superscripts in the same column are significantly different (*p* < 0.05). Control (chicken skin), sample A (PS at MR of 33–36 °C), sample B (PS at MR of 38–42 °C), sample C (PS at MR of 44–46 °C), sample D (PS at MR of 45–49 °C), and sample E (PS at MR of 55–60 °C).

**Table 3:** The textural properties and shear test force of the fat-replaced samples with palm shortenings (PS) of various melting ranges (MR) as compared to the control sample with chicken skin.

	Textural Properties				Shear Test Force	
Samples	Hardness (g)	Springiness (mm)	Cohesiveness	Chewiness (g/m^3^)	Maximum Shear Force (N)	Work of Shearing (N·s)
Control	10282.04 ± 880.05 ^b^	0.80 ± 0.01 ^a^	0.47 ± 0.01 ^a^	3872.04 ± 330.98 ^b^	4.82 ± 0.42 ^a^	60.93± 2.52 ^a^
A	5832.79 ± 731.54 ^c^	0.66 ± 0.01 ^bc^	0.37 ± 0.11 ^b^	1420.00 ± 223.01 ^c^	2.21 ± 0.15 ^b^	34.60 ± 2.58 ^b^
B	5432.25 ± 868.16 ^c^	0.63 ± 0.06 ^c^	0.36 ± 0.01 ^b^	1239.24 ± 319.20 ^c^	2.20 ± 0.46 ^b^	39.27 ± 4.36 ^b^
C	7075.28 ± 161.36 ^b^	0.66 ± 0.03 ^bc^	0.37 ± 0.04 ^b^	1731.49 ± 284.10 ^c^	2.18 ± 0.30 ^b^	32.00 ± 1.33 ^b^
D	10869.03 ± 1193.68 ^b^	0.73 ± 0.02 ^ab^	0.45 ± 0.00 ^a^	3603.66 ± 380.91 ^b^	3.35 ± 0.57 ^b^	35.02 ± 3.08 ^b^
E	15587.75 ± 1134.97 ^a^	0.75 ±0.02 ^a^	0.37 ± 0.04 ^b^	5551.95 ± 315.59 ^a^	4.79 ± 0.64 ^a^	59.64 ± 12.72 ^a^

Means followed by different uppercase superscripts in the same column are significantly different (*p* < 0.05). Control (chicken skin), sample A (PS at MR of 33–36 °C), sample B (PS at MR of 38–42 °C), sample C (PS at MR of 44–46 °C), sample D (PS at MR of 45–49 °C), and sample E (PS at MR of 55–60 °C).

**Table 4:** The proximate composition and colour properties of the fat-replaced samples with palm shortenings (PS) of various melting ranges (MR) as compared to the control sample with chicken skin.

	Proximate Composition				Colour Properties		
Samples	Moisture Content	Fat Content	Protein Content	Ash Content	L*	a*	b*
Chicken skin	53.10 ± 1.41	31.69 ± 1.82	13.65 ± 1.35	0.63 ± 0.10	-	-	-
Control	68.34 ± 1.00 ^a^	14.81 ± 0.85 ^bc^	14.52 ± 0.70 ^a^	1.49 ± 0.11 ^a^	56.73 ± 1.20 ^b^	2.74 ± 0.14 ^b^	9.65 ± 0.50 ^a^
A	62.47 ± 0.34 ^c^	19.28 ± 1.19 ^a^	15.50 ± 4.08 ^a^	1.49 ± 0.08 ^a^	58.88 ± 2.78 ^ab^	2.75 ± 0.10 ^b^	10.29 ± 0.83 ^a^
B	62.57 ± 0.67 ^c^	17.68 ± 0.58 ^ab^	14.06 ± 1.02 ^a^	1.36 ± 0.08 ^a^	58.39 ± 0.53 ^ab^	2.74 ± 0.14 ^b^	10.08 ± 0.16 ^a^
C	62.71 ± 0.10 ^c^	17.96 ± 0.33 ^a^	15.62 ± 0.75 ^a^	1.39 ± 0.08 ^a^	62.60 ± 1.43 ^a^	2.42 ± 0.13 ^bc^	10.97 ± 0.66 ^a^
D	60.56 ± 1.40 ^c^	20.29 ± 1.81 ^a^	15.77 ± 0.86 ^a^	1.36 ± 0.13 ^a^	60.93 ± 3.66 ^ab^	2.19 ± 0.07 ^c^	10.04 ± 1.28 ^a^
E	65.68 ± 0.54 ^b^	12.75 ± 1.40 ^c^	16.70 ± 2.30 ^a^	1.61 ± 0.20 ^a^	62.85 ± 0.80 ^a^	3.57 ± 0.16 ^a^	10.12 ± 0.14 ^a^

Means followed by different uppercase superscripts in the same column are significantly different (*p* < 0.05). Control (chicken skin), sample A (PS at MR of 33–36 °C), sample B (PS at MR of 38–42 °C), sample C (PS at MR of 44–46 °C), sample D (PS at MR of 45–49 °C), and sample E (PS at MR of 55–60 °C).

**Table 5:** The thermal denaturation temperatures and enthalpies of the fat-replaced samples with palm shortenings (PS) of various melting ranges (MR) as compared to the control sample with chicken skin.

	Peak 1				Peak 2			
Samples	Onset Temperature (°C)	Peak Temperature (°C)	End Temperature (°C)	Enthalpy (J/g)	Onset Temperature (°C)	Peak Temperature ( °C)	End Temperature (°C)	Enthalpy (J/g)
Control	35.01 ± 0.04 ^e^	36.28 ± 0.29 ^e^	37.28 ± 0.34 ^e^	0.15 ± 0.02 ^b^	62.28 ± 0.57 ^a^	74.29 ± 0.51 ^a^	85.18 ± 1.33 ^a^	0.13 ± 0.51 ^a^
A	33.18 ± 0.29 ^f^	34.77 ± 0.25 ^f^	36.61 ± 0.47 ^e^	0.14 ± 0.01 ^c^	62.61 ± 0.57 ^a^	72.21 ± 2.63 ^a^	81.75 ± 3.13 ^a^	0.15 ± 0.01 ^a^
B	38.13 ± 0.24 ^d^	40.87 ± 0.14 ^d^	41.84 ± 0.62 ^d^	0.22 ± 0.01.^c^	63.36 ± 1.67 ^a^	74.63 ± 0.54 ^a^	82.44 ± 1.42 ^a^	0.14 ± 0.03 ^a^
C	44.15 ± 0.59 ^c^	44.62 ± 0.81 ^c^	46.47 ± 0.42 ^c^	0.25 ± 0.11 ^c^	62.69 ± 2.52 ^a^	74.09 ± 0.43 ^a^	84.53 ± 1.28 ^a^	0.14 ± 0.02 ^a^
D	45.48 ± 0.40 ^b^	47.58 ± 0.66 ^b^	49.22 ± 0.61 ^b^	0.27. ± 0.04 ^b^	64.03 ± 1.95 ^a^	74.85 ± 0.45 ^a^	85.20 ± 1.60 ^a^	0.16 ± 0.17 ^a^
E	55.51 ± 0.19 ^a^	57.57 ± 0.28 ^a^	59.97 ± 0.75 ^a^	0.41 ± 0.07 ^a^	65.28 ± 2.87 ^a^	74.52 ± 1.45 ^a^	85.17 ± 0.07 ^a^	0.14 ± 0.01 ^a^

Means followed by different uppercase superscripts in the same column are significantly different (*p* < 0.05). Control (chicken skin), sample A (PS at MR of 33–36 °C), sample B (PS at MR of 38–42 °C), sample C (PS at MR of 44–46 °C), sample D (PS at MR of 45–49 °C), and sample E (PS at MR of 55–60 °C).

## Data Availability

Data is contained within the article.
